# A fluorescent hormone biosensor reveals the dynamics of jasmonate signalling in plants

**DOI:** 10.1038/ncomms7043

**Published:** 2015-01-16

**Authors:** Antoine Larrieu, Antony Champion, Jonathan Legrand, Julien Lavenus, David Mast, Géraldine Brunoud, Jaesung Oh, Soazig Guyomarc’h, Maxime Pizot, Edward E. Farmer, Colin Turnbull, Teva Vernoux, Malcolm J. Bennett, Laurent Laplaze

**Affiliations:** 1 Laboratoire de Reproduction et Développement des Plantes, CNRS, INRA, ENS Lyon, UCBL, Université de Lyon, 69364 Lyon, France; 2 Centre for Plant Integrative Biology, University of Nottingham, Nottingham LE12 5RD, UK; 3 Institut de Recherche pour le Développement, Unité Mixte de Recherche Diversité Adaptation et Développement des plantes, 911 Avenue Agropolis, 34394 Montpellier, France; 4 Laboratoire mixte international Adaptation des Plantes et microorganismes associés aux Stress Environnementaux, CP 18534 Dakar, Senegal; 5 Université Montpellier 2, Unité Mixte de Recherche Diversité Adaptation et Développement des plantes, 34394 Montpellier, France; 6 Department of Plant Molecular Biology, Université de Lausanne, 1015 Lausanne, Switzerland; 7 Department of Life Sciences, Imperial College London, London SW7 2AZ, UK

## Abstract

Activated forms of jasmonic acid (JA) are central signals coordinating plant responses to stresses, yet tools to analyse their spatial and temporal distribution are lacking. Here we describe a JA perception biosensor termed Jas9-VENUS that allows the quantification of dynamic changes in JA distribution in response to stress with high spatiotemporal sensitivity. We show that Jas9-VENUS abundance is dependent on bioactive JA isoforms, the COI1 co-receptor, a functional Jas motif and proteasome activity. We demonstrate the utility of Jas9-VENUS to analyse responses to JA *in planta* at a cellular scale, both quantitatively and dynamically. This included using Jas9-VENUS to determine the cotyledon-to-root JA signal velocities on wounding, revealing two distinct phases of JA activity in the root. Our results demonstrate the value of developing quantitative sensors such as Jas9-VENUS to provide high-resolution spatiotemporal data about hormone distribution in response to plant abiotic and biotic stresses.

## Abstract (web summary)

Jasmonate regulates multiple aspects of plant growth, development and stress responses. Here, Larrieu *et al*. develop a fluorescent biosensor that allows jasmonate perception to be monitored at previously unobtainable levels of spatiotemporal resolution in *Arabidopsis*.

## Graphical abstract

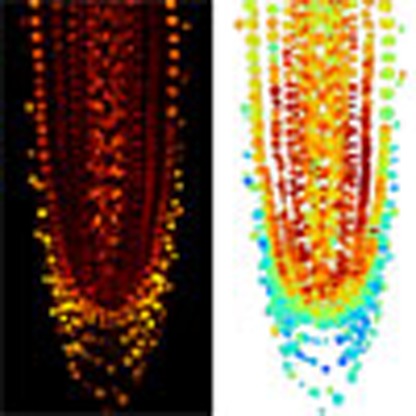

Plants are subject to a variety of biotic and abiotic stresses such as the presence of pathogens, insect or mechanical injury, and several environmental stresses. These stresses lead to changes in hormone levels and gene expression that occur within minutes and activate local and systemic responses. Many of these adaptive responses are regulated by the plant hormone jasmonic acid (JA) and its derivatives, collectively referred to as jasmonates^1^. However, due to the current lack of tools to visualize the sites of JA perception *in planta*, there are major gaps in our understanding of how JA exerts its effects locally or systemically. JA signalling triggers large-scale changes in gene expression^2^. In cells with a low JA content, transcription factors that regulate JA-responsive genes are repressed by members of the Jasmonate-ZIM-Domain (JAZ) protein family. The bioactive form of JA conjugated to isoleucine, termed JA-Ile, promotes the binding of JAZ repressors to the F-box protein CORONATINE INSENSITIVE1 (COI1). This F-box protein is part of the SCF^COI1^ E_3_ ubiquitin ligase complex that promotes JAZ degradation via the ubiquitin/26S proteasome pathway. Therefore, in the presence of JA-Ile, JAZ proteins are degraded and transcription factors are relieved from repression^3^,^4^,^5^. A specific Jas motif in JAZ proteins is responsible for their interaction with COI1 and their JA-dependent degradation^6^,^7^.

Biosensors are tools that transform a recognition event such as the perception of a small molecule by a receptor into a signal that can be easily detected and quantified^8^,^9^. In theory, a biosensor should (1) respond specifically to its target, (2) demonstrate a quantitative response over a physiologically relevant concentration range, (3) provide a dynamic and spatially resolved response *in vivo* and (4) have no impact on the endogenous signalling system it is reporting^8^,^9^. The recent development of biosensors for phytohormones such as auxin or abscisic acid (ABA) has led to significant progress in our understanding of the distribution of these molecules and their signalling mechanisms^10^,^11^,^12^. Here we describe a novel JA perception biosensor termed Jas9-Venus and demonstrate its utility to quantitatively and dynamically analyse JA response and distribution *in planta* at the cellular scale.

## Results

### Jas9-VENUS is a biosensor for perception of bioactive JA

**Figure 1:**
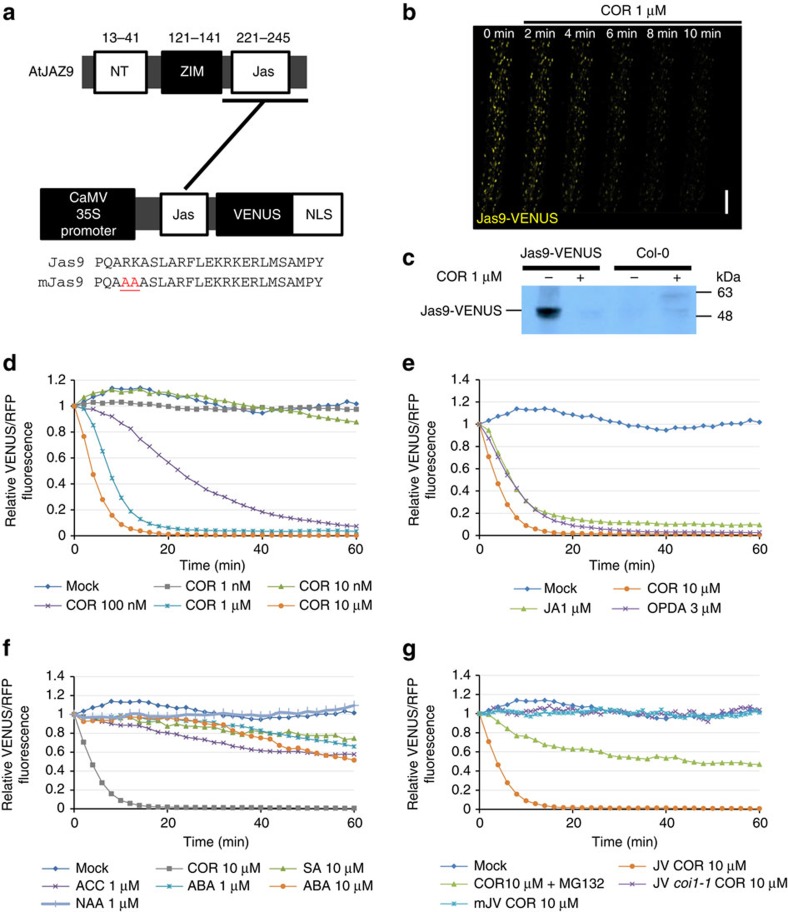
Jas9-VENUS is a biosensor for bioactive JA perception. (**a**) Schematic representation of the *AtJAZ9* gene, the Jas9-VENUS and mJas9-VENUS constructs, as well as the protein sequences of their respective Jas motif (Jas, Jas motif; NLS, nuclear localization signal; NT, amino terminus; ZIM, ZIM motif). Numbers above the schematic represent amino acid position. (**b**) Time-course confocal laser scanning micrographs of Jas9-VENUS at the indicated time points after treatments with 1 μM coronatine (COR; scale bar, 100 μm). (**c**) Western blot analysis of total protein extracts of Jas9-VENUS and Col-0 seedlings treated for 30 min with or without 1 μM COR and probed with an anti-GFP antibody (uncropped western blotting is shown in Supplementary Fig. 1d). (**d**) Time-course quantification of Jas9-VENUS fluorescence, normalized to H2B-RFP fluorescence, in response to various concentrations of coronatine (*n*=3). (**e**) Time-course quantification of Jas9-VENUS fluorescence, normalized to H2B-RFP fluorescence, in response to JA-Ile precursors JA and OPDA (*n*=2). (**f**) Time-course quantification of Jas9-VENUS fluorescence, normalized to H2B-RFP fluorescence, in response to auxin (α-naphthalene acetic acid (NAA)), ABA, ACC (ethylene precursor) or SA (*n*=2). (**g**) Time-course quantification of Jas9-VENUS fluorescence, normalized to H2B-RFP fluorescence, in response to treatments with COR in a WT or a *coi1-1* mutant, in response to COR and MG132 (proteasome inhibitor) treatments, and of mJas9-VENUS fluorescence, normalized to H2B-RFP fluorescence, in response to COR (*n*=2). All the data shown derive from a representative experiment and the number of replications of each experiment is indicated. JV, Jas9-VENUS; mJV, mJas9-VENUS.

Previous studies have shown that JAZ proteins such as JAI3 and JAZ1 are degraded by the proteasome pathway in response to JA, and that this degradation is dependent on a specific Jas motif in the protein^3^,^4^. This provided a potential mechanism to develop biosensors to follow JA perception *in vivo*. To test this hypothesis, we generated translational fusions between the putative Jas motif of AtJAZ1 and AtJAZ9 proteins, including key amino acid residues on either side of the Jas motif^6^, the fast maturing VENUS variant of the yellow fluorescent protein^13^,^14^ and a N7 nuclear localization signal^15^ (Fig. 1a). The corresponding constructs were expressed under the control of the constitutive CaMV 35S promoter and transformed into *Arabidopsis thaliana* plants expressing a nuclear Histone H2B marker fused to the red fluorescent protein (H2B-RFP), under the regulation of the same promoter, for ratiometric measurements^16^ (Supplementary Fig. 1a–c). This approach limits biases in fluorescence levels that would be due to altered regulation of the CaMV 35S promoter. Robust fluorescence was only observed for Jas^JAZ9^-VENUS lines, suggesting that Jas^JAZ1^-VENUS turnover is faster than its maturation time. This is consistent with the fast turnover observed for a JAZ1-GUS fusion^4^. We therefore focused our studies on Jas^JAZ9^-VENUS, henceforth called Jas9-VENUS.

Next, we probed the quantitative relationship between Jas9-VENUS and JAs. We analysed changes in fluorescence in transgenic seedlings following JA treatment, using confocal microscopy. The bacterial toxin coronatine is structurally similar to JA-Ile and is a potent agonist of the JA receptor^7^. We observed that low levels of coronatine led to rapid degradation of Jas9-VENUS in plant roots (Fig. 1b,d, Supplementary Fig. 1d and Supplementary Movie 1). Time-course imaging of roots revealed that the Jas9-VENUS signal started to decrease within minutes after coronatine treatments. Western blot analyses confirmed that the decrease in fluorescence was due to Jas9-VENUS degradation (Fig. 1c). Fluorescence quantification indicated that Jas9-VENUS degradation was dose dependent (Fig. 1d) and specifically induced by bioactive JAs (Fig. 1e) but not, or very delayed and only to a limited degree, by other hormones such as auxin, ABA, ethylene (ACC (1-aminocyclopropanecarboxylic acid), ethylene precursor) or salicylic acid (SA) (Fig. 1f). The limited and delayed degradation of Jas9-VENUS observed on treatment with other hormones is likely to result from cross-talk between these signals and their transduction pathways with JA signalling^17^. The JA-Ile precursors, JA and 12-oxo-phytodienoic acid (OPDA) triggered a very rapid response, indicating that these molecules quickly enter the cell and get metabolized to generate JA-Ile (Fig. 1e). Moreover, degradation of Jas9-VENUS was abolished in the *coi1-1* mutant background^18^ and strongly reduced when plants were treated with the proteasome inhibitor MG132 (Fig. 1g). A stabilized Jas9-VENUS version was created by site-directed mutagenesis on the critical amino acids R and K at position 223 and 224, respectively, of the Jas motif being substituted with alanine residues. These mutations have been demonstrated to prevent binding to COI1, and therefore JAZ proteins degradation in response to JA treatments^19^. This mutated version of the sensor, termed mJas9-VENUS, was not degraded in response to coronatine treatment (Fig. 1g). Altogether, these data indicate that Jas9-VENUS is a quantitative and specific sensor of jasmonate perception *in planta*.

Next, we compared the growth response of Jas9-VENUS and control (Pro_35S_:H2B-RFP) plants to coronatine. Dose–response curves revealed that Jas9-VENUS roots exhibit the same profile of root growth inhibition by coronatine as control plants (Supplementary Fig. 2a). Similarly, the induction of JA-responsive genes in response to wounding was maintained in Jas9-VENUS plants (Supplementary Fig. 2b). Hence, our data indicate that the Jas9-VENUS sensor has no detectable effect on either physiological or molecular responses to JA.

### Use of Jas9-VENUS to map local changes in JA distribution

**Figure 2:**
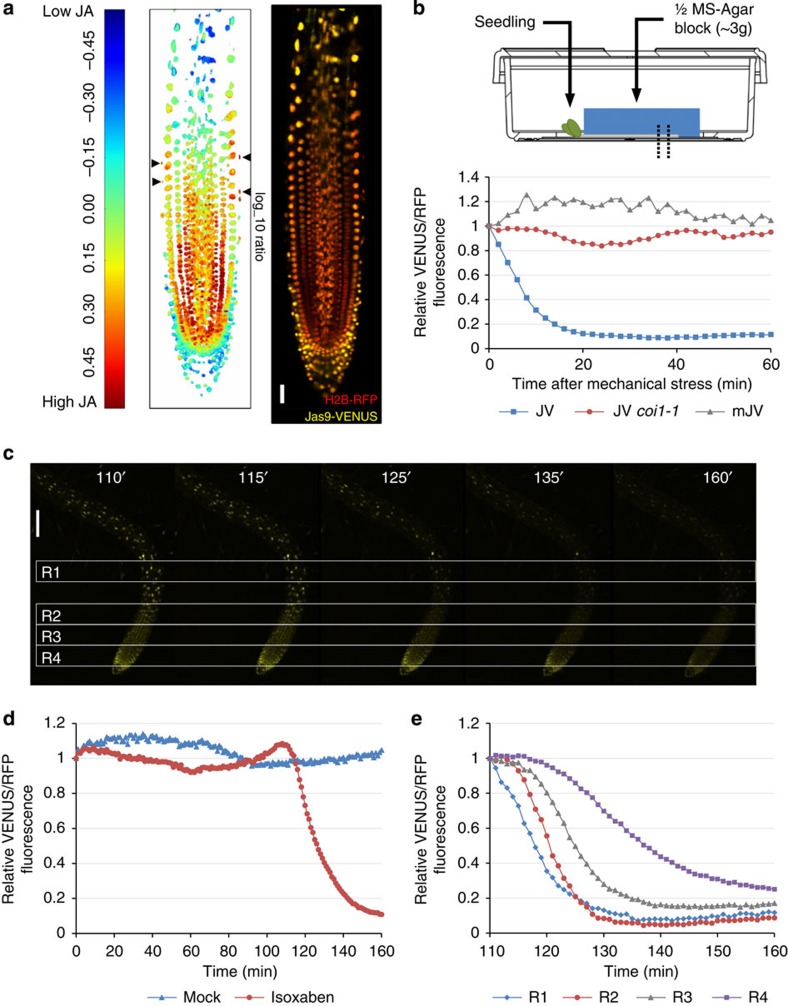
Jas9-VENUS can be used to map local changes in bioactive JA. (**a**) Distribution map of bioactive JA in the root apical meristem based on the ratio of Jas9-VENUS fluorescence to H2B-RFP fluorescence of a representative seedling. The overlay of the Jas9-VENUS and H2B-RFP channels used for generating the root map is shown (scale, 30 μm). In total, three independent transgenic lines were analysed (*n*=5) and representative maps are shown in Supplementary Fig. 3. The map revealed that basal JA levels are higher in the epidermal, ground tissue, pericycle and vascular initials, and in their daughter cells in the division zone than in other parts of the root. (**b**) Seedlings are mechanically stimulated by gently laying down a block of agar (~3 g) on the entire root. Time-course quantification of Jas9-VENUS fluorescence, normalized to H2B-RFP fluorescence, in a WT or *coi1-1* mutants, and of mJas9-VENUS fluorescence, normalized to H2B-RFP fluorescence, in response to a mechanical stimulus. (**c**) Confocal micrographs taken at the indicated time points after treatments with 10 μM isoxaben (scale bar, 100 μm, *n*=2). (**d**) Time-course quantification of Jas9-VENUS fluorescence, normalized to H2B-RFP fluorescence, over 160 min after isoxaben and mock treatments. (**e**) Time-course quantification of Jas9-VENUS fluorescence, normalized to H2B-RFP fluorescence, between 110 and 160 min after treatments with isoxaben for the four regions of the root indicated on the micrographs. Drawing used in **b** kindly provided by Greiner Bio-One GmbH, Frickenhausen, Germany. All the data shown derive from a representative experiment and the number of replications of each experiment is indicated. JV, Jas9-VENUS; mJV, mJas9-VENUS.

JA inhibits root growth, notably through the direct repression of *PLETHORA* (*PLT*) genes by the transcription factor MYC2 (ref. 20). However, the spatial distribution of JA in the root meristem remains unclear. We used Jas9-VENUS and H2B-RFP fluorescence to generate a map of JA distribution in the root apical meristem (Fig. 2a, Supplementary Fig. 3 and Supplementary Data 1). As Jas9-VENUS degradation is COI1 dependent, we first analysed the expression of *COI1* in the root using a translational Pro_COI1_:COI1-VENUS fusion. The construct revealed a homogenous distribution of the receptor in the majority of root cell types (Supplementary Fig. 4 and Supplementary Data 1). Slightly higher expression was detected in elongating epidermal cells and these cells might therefore be more sensitive to JA. However, the spatial pattern of Jas9-VENUS fluorescence that we observed in the root apical meristem should not be influenced by differences in JA perception, except in the mature epidermis, where potential overestimation of JA levels occurs due to high expression of *COI1*. Our imaging studies revealed that basal JA levels are higher in the epidermal, ground tissue, pericycle and vascular initials, and in their daughter cells in the division zone than in other parts of the root (Fig. 2a and Supplementary Fig. 3). Interestingly, a root JA gradient was revealed with highest concentrations close to the tip, which decreased as cells move away from the stem cell niche. Low JA accumulation occurred in the quiescent centre. Considering that jasmonate is a stress-related hormone present at low concentrations in unstimulated tissues^21^, this suggests a role for JA in the functioning of the root apical meristem. No JA was found to accumulate in the root cap, except in last two cells of the lateral root cap. Hence, Jas9-VENUS together with the COI1 translational reporter can be used to map the distribution of JA in a given cell, tissue or organ.

Mechanical stress is also known to trigger JA production in plants^21^. Therefore, we investigated the dynamics of local response of the Jas9-VENUS sensor to mechanical stress by applying a mechanical pressure to roots using an agar block. The reporter was degraded in all of the cells under pressure in <20 min, indicating that JA signalling was rapidly activated throughout the root (Fig. 2b). The degradation was again COI1 dependent as demonstrated by the lack of response in a *coi1-1* mutant background or in the mJas9-VENUS line (Fig. 2b). Similarly, perturbations in cell wall properties by genetic (mutations) or pharmacological (isoxaben) inhibition of cellulose biosynthesis lead to mechanical stress and JA production^22^,^23^. Consistently, we observed a rapid degradation of the Jas9-VENUS sensor starting in the elongation zone around 110 min after isoxaben treatment and then moving to neighbouring regions of the root apex (Fig. 2c–e and Supplementary Movie 2). Hence, the Jas9-VENUS sensor reveals JA-Ile perception at a cellular resolution in plant roots in response to local stresses and allows the spread of the JA signal to be monitored at the tissue level.

### Use of Jas9-VENUS to study long-distance JA signalling

**Figure 3:**
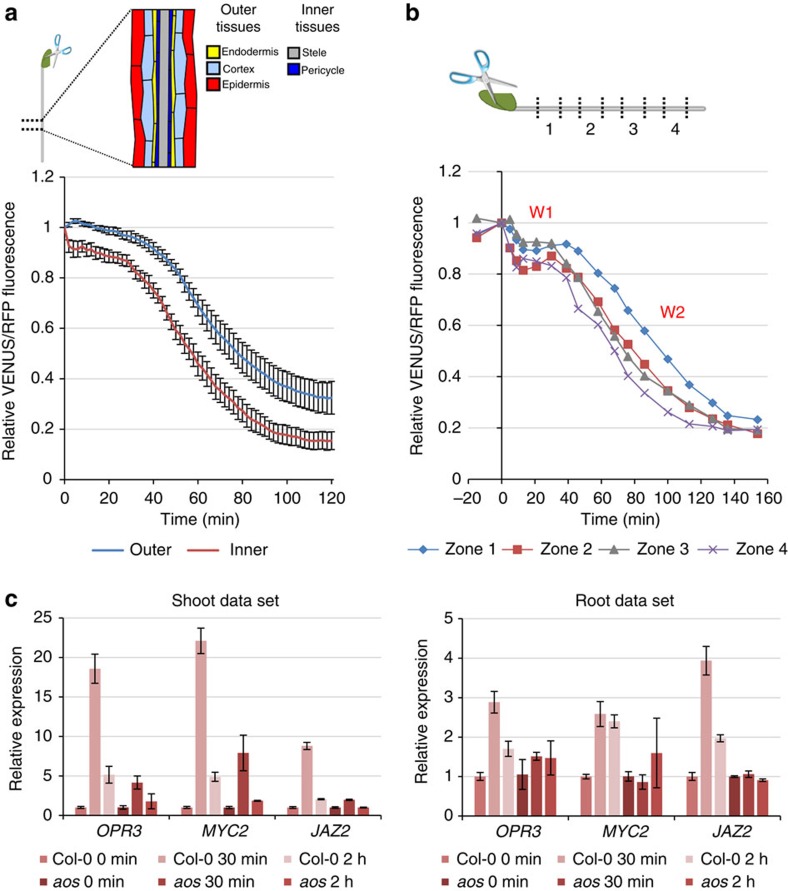
Jas9-VENUS can be used to study long-distance JA signalling *in planta*. (**a**) Seedlings were wounded by cutting a cotyledon using dissection scissors. Time-course quantification of Jas9-VENUS fluorescence, normalized to H2B-RFP fluorescence, in the inner and outer tissues of the root over 120 min after wounding. Error bars show the s.e.m. of 13 replicates. (**b**) Time-course quantification of Jas9-VENUS fluorescence, normalized to H2B-RFP fluorescence, in the inner tissues at four positions along the root (indicated on the diagram) before and after wounding. (**c**) Shoots and roots of 1-week-old WT or *aos* seedlings were harvested 30 min and 2 h after wounding and the expression of wound-inducible genes were investigated using RT–qPCR. The experiment was repeated twice and the results show a representative data set. Error bars represent the s.d. of four technical replicates.

The mechanisms by which plants perceive and respond to tissue injury are complex. Biotic or mechanical stresses lead to systemic induction of host defence responses that protects healthy tissues from secondary attack. This JA-mediated process involves long-distance communication between tissues^24^ and occurs within minutes of tissue injury. To analyse long-distance JA signalling, we measured VENUS fluorescence in the root following wounding of one cotyledon. We observed a rapid decrease in Jas9-VENUS fluorescence in the root (Fig. 3a, Supplementary Fig. 5 and Supplementary Movie 3). In contrast to local mechanical stress, the decrease in VENUS fluorescence occurred in two distinct phases in 10 of 14 experiments (Fig. 3a and Supplementary Fig. 5). An initial phase amounting to a loss of ~8% of the signal was completed ~2 min after wounding (Fig. 3a). This first phase was mainly detected in the stele, suggesting that the signal was moving in the vasculature (Fig. 3a and Supplementary Fig. 5). To determine the velocity of the signal triggered by wounding, fluorescence was measured at four different positions along the root (Fig. 3b). Interestingly, the loss of VENUS fluorescence characteristic of this first phase occurred simultaneously at the root/shoot junction (Zone 1) and in close vicinity of the root tip (Zone 4), which were 1 cm apart. Based on the experiment set-up, we estimate the speed of this first wave to exceed 1 cm min^−1^. This observation is in the same order of magnitude as measurements reported for leaf-to-leaf wound signalling^25^. A second phase observed in all experiments started at ~30 min and lasted for 90 min, leading to a further 70% decrease of the fluorescence signal. It occurred first in the stele and then in the outer tissues, and starts in the root tip region (Zone 4; Fig. 3a and Supplementary Fig. 5). Using reverse transcriptase quantitative–PCR (RT–qPCR), we confirmed that the decrease in VENUS fluorescence after wounding was not due to an altered expression of the receptor *COI1* and of the sensor *Jas9-VENUS*, as the expression of both mRNA is stable after wounding in the shoot and in the root (Supplementary Fig. 6a,b). We also showed that the reduction in VENUS fluorescence in response to wounding required a functional Jas motif (Supplementary Fig. 7). Thus, the Jas9-VENUS reporter revealed that a two-step JA response is induced in the root in response to wounding in the aerial part of the plant and allows the estimation of the speed of the signal triggering this response.

We investigated whether the response observed with the Jas9-VENUS sensor could be correlated with documented JA responses. Roots and aerial parts from *Arabidopsis* plants were harvested before and 30 min and 2 h after wounding a cotyledon, and the expression of genes induced by JA was measured by RT–qPCR. We observed a rapid induction of JA-responsive genes in roots after wounding in accordance with the rapid response of the Jas9-VENUS reporter (Fig. 3c). This induction is dependent on JA production, as the biosynthesis mutant *aos*^26^ exhibited little or no induction of those genes (Fig. 3c). Moreover, JA was recently shown to stabilize the JA-responsive MYC2 transcription factor^27^. Accordingly, we observed that a stabilization of a MYC2-GFP fusion protein in the root 1 h after wounding the cotyledon (Supplementary Fig. 8). This stabilization was lost in a MYC2ΔDE-GFP construct that was shown to be unable to respond to JA^27^. This is consistent with an induction of local JA production in the root corresponding to the second phase of the response detected with Jas9-VENUS. Altogether, gene expression data and MYC2 protein stabilization are consistent with the dynamics of JA signalling that we observed with the Jas9-VENUS sensor.

## Discussion

Here we describe Jas9-VENUS, a new tool to monitor the perception of biologically active jasmonates *in planta*. We show that Jas9-VENUS is specifically and rapidly degraded in the presence of bioactive JA in a dose-dependent manner. Moreover, we demonstrate that Jas9-VENUS degradation is dependent on COI1 and the proteasome. Finally, our results indicate that the Jas9-VENUS construct does not perturb JA perception and responses. Altogether, Jas9-VENUS has all the attributes of a JA perception biosensor, that is, its fluorescence is directly correlated to the combinatorial action of the signal and the receptor machinery.

Our results suggest that Jas9 might be more stable than other Jas motifs such as Jas1 and might therefore not be as responsive for fast JA responses or suitable to calculate velocity for propagation of rapid JA responses. However, in practice, Jas9-VENUS degradation was dose dependent and responded very rapidly (in a few minutes) to JA. Moreover, analyses of JA-responsive gene expression are consistent with the observed dynamics of Jas9-VENUS degradation. Jas9-VENUS thus offers a good compromise between a strong fluorescence signal, sensitivity, specificity and a quantitative and rapid response, as expected from a genetically encoded sensor^9^. In addition, we used of a ratiometric approach combining the Jas9-VENUS construct to a nuclear-localized 35S-H2B:RFP construct, to prevent errors due to a transcriptional effect on the 35S promoter. However, we cannot rule out that the use of two different constructs containing the 35S promoter could still induce biases in ratio quantification due to genomic position effects that might have an impact on the expression of the transgene or to silencing. Ideally, both Jas9-Venus and H2B-RFP would be expressed from the same promoter as a single protein that would be cleaved to avoid these problems^28^. Such a design feature would prove useful in future biosensors development.

Our studies of local and long-distance JA responses showed the value of Jas9-VENUS to image the quantitative dynamics of these responses at a high temporal and spatial resolution. Using Jas9-VENUS, we were able to generate a map of JA accumulation in the root apical meristem. Exogenous JA is known to regulate root growth^1^ and jasmonates could mediate root response to various biotic and abiotic environmental signals. Here we show that JA accumulates in the epidermis, ground tissues and vascular tissue from the initials to the end of the division zone in undamaged plants. This is coherent with the expression of the JA-inducible *ASA1* gene in the root tip^29^. *ASA1* encodes an enzyme involved in auxin biosynthesis and this JA-auxin cross-talk could mediate some of the JA-regulated root responses to environmental signals. Moreover, our map revealed JA accumulation in the outer lateral root cap and in the epidermis. This is in agreement with previously reported map of JA-related gene expression in the root apical meristem^30^, suggesting that JA distribution might be the primary determinant of JA-induced gene expression in this tissue. Interestingly, outer lateral root cap cells were shown to enter programmed cell death^31^. Although the role of JA in programmed cell death has been documented in other systems^32^, this suggests a novel role for JA in regulating lateral root cap development. Finally, the high levels of JA detected in the ground tissue initials and daughter cells suggest an unanticipated role for JA in the activity of the root apical meristem.

We also used the Jas9-VENUS sensor to analyse long-distance signalling between the aerial parts and the root following a wound. It revealed that a two-step JA response is induced in the root in response to wounding in the aerial part of the plant. We first observed a rapid signal (>1 cm min^−1^) moving through the stele and inducing a limited degradation of Jas9-VENUS presumably through a limited JA production. This first phase was relatively weak and localized in the stele but robust enough to be detected in 10 out of 14 experiments. Moreover, the variability of response we observed could also result from the difficulties of performing wounding in a reproducible way. In leaves, this fast-moving signal is probably related to the membrane depolarization occurring in sieve elements in response to wounding and responsible for leaf-to-leaf signalling^25^,^33^. A similar mechanism may participate in leaf-to-root signalling and might induce the very rapid conversion of a local pool of inactive jasmonate precursors into active JA. This would in turn trigger the expression of JA biosynthesis genes and therefore strongly enhance JA accumulation that would contribute to the second phase of the response. This biphasic mechanism would therefore serve to amplify an initial rapid systemic signal to induce a later, stronger local response in the root. Electrical activity in the vasculature is probably not the only source of signals that lead to jasmonate production in wounded leaves^25^ and it is possible that other mechanisms underlie the second, stronger phase of jasmonate signalling that we observed in roots. Importantly, our results suggest that wounding not only induces systemic defence in the leaves but is also likely to modulate root development, physiology and defence responses.

Jasmonate is a fundamental regulator of plant responses to various abiotic and biotic stresses. The Jas9-VENUS sensor paves the way to a better characterization of the JA signalling pathway *in planta*. Our results further illustrate how fluorescent sensors can be used to perform multi-scale analyses of the distribution of small signalling molecules.

## Methods

### Chemicals and hormones

Coronatine, JA, SA, α-naphthalene acetic acid, ACC (ethylene precursor), (±)-ABA, MG132 and isoxaben were purchased from Sigma. OPDA was purchased from Cayman Chemical (Keystone, Colorado, USA). Coronatine, SA, α-naphthalene acetic acid, MG132 and isoxaben were dissolved in 100% dimethyl sulfoxide, ACC was dissolved in double distilled H_2_O, ABA in 100% methanol and JA in 100% ethanol. All compounds were dissolved to a stock concentration of 50 or 100 mM. OPDA was purchased diluted in 100% ethanol (100 μg per 100 μl~3.4 mM).

### *Arabidopsis* lines

The JA biosynthetic mutant *aos*^26^ and the JA response mutant *coi1-1* (ref. 18) were obtained from the Nottingham Arabidopsis Centre. The p35S:H2B-RFP, p35S:MYC2-GFP and p35S:MYC2ΔDE-GFP line were described previously^16^,^27^.

### Seed sterilization and plant growth

Seeds were surface sterilized for 5 min in 10% bleach, 0.1% Triton X-100, then washed three times with sterile double distilled H_2_O. Seeds were stratified at 4° for 2 days to synchronize germination. For time-lapse imaging on the confocal microscope (treatments and wound responses), sterile seeds were sown directly onto glass-bottom petri dishes (ref 627861 from Greiner (Germany), and ref 3930–035 from Iwaki (Japan)) against a block of ½ Murashige and Skoog medium (2.17 g MS salts per litre (Sigma)) at pH 5.7 solidified with 1% bacto-agar (Appleton Woods; as shown in Supplementary Fig. 9). Seedlings were grown under continuous light (100 μE m^−2^ s^−1^). For all other experiments (mechanical stress, bulk up and gene expression), sterile seeds were sown onto round or square petri dishes containing MS agar media as described above.

### Jas9-VENUS constructs

The Jas motif from *AtJAZ9* was amplified using primer JAZ9-F2 and JAZ9-R1 by PCR using complementary DNA produced from 2-week-old *Arabidopsis* plants treated with 50 μM JA during 1 h. The mutated version of the Jas9-VENUS sensor was generated by PCR using primers JAZ9-F3m and JAZ9-R3m. PCR products were cloned into pENTR-D TOPO (Invitrogen). The Jas motif sequence or the mutated version were then fused in-frame to VENUS-N7 under the control of the cauliflower mosaic virus 35S promoter in the pH7m34GW binary plasmid^34^, using the Multisite Gateway three-fragment vector construction kit (Invitrogen). Plasmid constructs were confirmed by sequencing. The vectors were transformed into *Agrobacterium tumefaciens* strain GV3101 and into *Arabidopsis* p35S-H2B:RFP (Col-0 ecotype) using the floral dip transformation protocol. Lines with single insertions were selected based on the segregation of antibiotic resistance and fluorescence. Line 6 was used in most experiments unless specifically stated.

### COI1 constructs

*COI1* genomic sequence and its promoter were amplified by PCR using COI1-Fw and COI1-Rev-Full primers. The PCR product was purified and cloned into the entry clone pENTR-D-TOPO using TOPO cloning (Invitrogen), according to the manufacturer’s instructions. The fast-maturing yellow fluorescent protein VENUS was added immediately before the STOP codon using multi-site gateway cloning into the destination vector pK7m34GW, to create the binary vector pK7m34GWpCOI1-COI1-VENUS. The vector was transformed into *Agrobacterium* strain GV3101 and into *Arabidopsis* Col-0 ecotype using the floral dip transformation protocol^35^.

### *Arabidopsis* protein extraction

Extraction of soluble proteins was performed on 1-week-old seedlings grown on ½ MS+agar plates. Seedlings were treated continuously with liquid ½MS medium for 30 min with 1 μM coronatine or with dimethyl sulfoxide (Mock). Seedlings were harvested, frozen in liquid nitrogen and then ground using a Qiagen Tissue Lyser II (30 Hz during 30 s). The powder was resuspended in SDG buffer (0.0625 M Tris-HCl pH6.8, 2.5% SDS, 2% dithiothreitol (0.13 M), 20% glycerol) and subjected to three cycles of freezing/thawing in liquid nitrogen. Proteins were boiled for 5 min at 95 °C, followed by two successive rounds of centrifugation (20 min, 18,000 *g* at 4 °C then 5 min, 18,000 *g* at 4 °C) to remove cell debris.

### Western blotting

The samples were boiled for 5 min at 95 °C before being loaded onto a gel (10 μl per lane). SDS–PAGE was performed on 10% polyacrylamide gel. After transfer onto a nitrocellulose membrane, protein amounts in each lane were checked using Ponceau staining. Immunoblotting analysis was realized using a primary rabbit polyclonal anti-GFP antibody (ab290, Abcam, diluted 1:5,000) and a secondary anti-rabbit Ig-HRP (NA934-1ML, Amersham, diluted 1:10,000). Proteins were visualized using the enhanced chemiluminescence kit (Amersham).

### Confocal microscopy

Jas9-VENUS seedlings were imaged on an inverted Leica SP5 confocal laser scanning microscope (Leica Microsystems, Germany) or an inverted Nikon Eclipse Ti-U confocal microscope (Nikon, Japan). Scanner and detectors settings used for one experiment were optimized to avoid saturation and to maximize resolution and kept unchanged throughout the experiment. VENUS was excited using the 514 nm line (Leica SP5) or the 488 nm line (Nikon Eclipse) of an argon laser. VENUS fluorescence was collected from 520 to 560 nm (using the AOBS of the SP5) or using a 515/30 detector (Nikon Eclipse). RFP was excited using a 561-nm laser diode (SP5) or the 543-nm line of a Green He/Ne laser (Nikon Eclipse). RFP fluorescence was collected from 590 to 680 nm (using the AOBS of the SP5) or using a 605/75 detector (Nikon Eclipse).

Images were taken with a fixed delay of 2 min over a minimum time of 1 h using the × 10 objective, to maximize the number of nuclei being observed. Initially, seedlings were imaged following the protocol described^16^. Briefly, seedlings were grown onto ½ MS-agar round or square petri dishes and gently transferred onto glass-bottom petri dishes. A small block of ½ MS agar was slowly dropped onto the seedling and fluorescence was followed over time just above the elongation zone of the root. Initial experiments showed that such treatment induced the degradation of the Jas9-VENUS reporter. Subsequently, seeds were directly germinated onto the glass-bottom petri dishes (as shown on Supplementary Fig. 9) and roots that grew in between the agar block and the glass bottom were imaged. In such configuration, a stable VENUS fluorescence could be followed over time in the absence of treatments, although the signal fluctuated over time (±20%). Treatments were provided by adding liquid ½ MS solution (+ indicated chemicals) at the same temperature next to the seedling that was going to be imaged. Mock treatments resulted in no major changes in fluorescence (comparable to no addition of liquid medium).

Other markers (pCOI1:COI1-VENUS, p35S:MYC2-GFP and p35S:MYC2ΔDE-GFP) and images for the JA and COI1 root maps were imaged on an inverted Zeiss 710 confocal laser scanning microscope. Scanner and detector settings used for one experiment were optimized to avoid saturation and to maximize resolution, and kept unchanged throughout the experiment. VENUS was excited using the 514-nm line of an argon laser and collected from 520 to 560 nm. Green fluorescent protein (GFP) was excited using a 488-nm line of an argon laser and collected from 495 to 550 nm.

### Fluorescence quantification

Fluorescence quantification was performed as described^36^. Briefly, background fluorescence was removed using a threshold and only fluorescence coming from the nuclei was quantified. Plots presented show changes in raw integrated density values over time, measured using FIJI software. Ratios (raw integrated density of the VENUS channel divided by the raw integrated density of the red channel) were calculated using Microsoft Excel software.

### JA and COI1 root maps

The acquired confocal images were imported for processing using Python programming language. Images with the lowest saturation possible were used to avoid computational artefacts and to preserve a good signal-to-noise ratio. Background (shown in white) was removed for the JA root maps by thresholding the H2B-RFP fluorescence to an intensity above 34 and for the COI1 root map by thresholding the COI1-VENUS fluorescence to an intensity above 16. For the JA root map, the ratiometric computation is the ratio of Jas9-VENUS/H2B-RFP fluorescence. To enhance the ‘high JA’ and ‘low JA’ differences, the obtained ratiometric values were transformed into a log_10_ scale. For the COI1 root map, the COI1-VENUS fluorescence values were centred around the mean to enhance for differences in fluorescence.

### Reverse transcriptase quantitative–PCR

One-week-old seedlings were wounded by cutting a cotyledon. RNA was extracted from shoots and root separately using the Spectrum Plant Total RNA kit (Sigma). Poly(dT) cDNA was prepared from 1 μg of total RNA with Superscript III reverse transcriptase (Invitrogen) and analysed on a StepOnePlus apparatus (Life Technologies) with the SYBR Green PCR Master Mix (Applied Biosystem), according to the manufacturer’s instructions. Targets genes were quantified with specific primer pairs designed with the Universal Probe Library Assay Design Center (Roche Applied Science). All reactions were done in quadruplicate and data were analysed with Microsoft Excel 2007 (Microsoft Corporation, Redmond, USA). Expression levels were normalized to At1G04850 (CTRL1) and calibrated to unwounded seedling using the ΔΔCt method.

## Additional information

**How to cite this article:** Larrieu, A. *et al*. A fluorescent hormone biosensor reveals the dynamics of jasmonate signalling in plants. *Nat. Commun.* 6:6043 doi: 10.1038/ncomms7043 (2015).

## Supplementary Material

Supplementary Figures and Table Supplementary Figures 1-9 and Supplementary Table 1

Supplementary Movie 1 Time course showing the Jas9-VENUS and H2B-RFP channels in response to 1µM coronatine. Degradation of the Jas9-VENUS protein occurs within minutes after coronatine treatment.

Supplementary Movie 2 Time course showing the Jas9-VENUS and H2B-RFP channels in response to 10µM isoxaben. Degradation of the Jas9-VENUS protein occurs with a two-hour delay after treatment and starts in the elongation zone before spreading to the root apex.

Supplementary Movie 3 Time course showing the Jas9-VENUS and H2B-RFP channels in response to a wound (cut in a cotyledon). Degradation of the Jas9-VENUS protein occurs in two phases. A first, rapid response leads to a 10% reduction of fluorescence primarily in the vascular tissues and a second, more delayed response, leads to a loss of 80-90% of fluorescence 45 minutes after wounding.

Supplementary Data 1 Python scripts used to generate the JA and the COI1 root maps.
