# Corrigendum: A fluorescent hormone biosensor reveals the dynamics of jasmonate signalling in plants

**DOI:** 10.1038/ncomms10704

**Published:** 2016-02-10

**Authors:** Antoine Larrieu, Antony Champion, Jonathan Legrand, Julien Lavenus, David Mast, Géraldine Brunoud, Jaesung Oh, Soazig Guyomarc′h, Maxime Pizot, Edward E. Farmer, Colin Turnbull, Teva Vernoux, Malcolm J. Bennett, Laurent Laplaze

Nature Communications 6: Article number: 604310.1038/ncomms7043 (2015); Published: 01162015; Updated: 02102016.

It has come to our attention that the control version of the Jas9-VENUS reporter construct reported in this Article, mJas9-VENUS, which consists of a mutated version of Jas9 fused to VENUS, does not contain a nuclear localization signal as described in Fig. 1a. The conclusion that degradation of the Jas9-VENUS reporter is dependent on jasmonate signalling in the nucleus remains unaffected by this error, as the reporter construct itself contains a nuclear localization signal, and the mJas9-VENUS control construct also localizes to the nucleus, even in the absence of this sequence (Fig. 4). As a result we do not foresee any practical implications for the use of either the Jas9-VENUS sensor construct or the mJas9-VENUS control.

**Figure 4 | mJas9-VENUS localizes to the nucleus in the mature part of the root.**


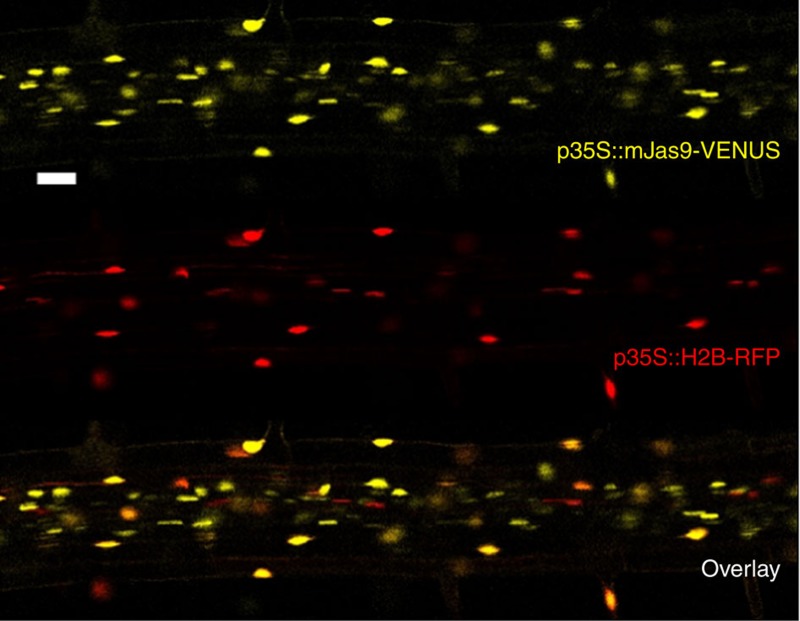
Images show the mature root of a 5-day-old Arabidopsis seedling expressing the nuclear marker H2B-RFP and the mJas9-VENUS control reporter construct. Scale bar, 20 mm.

