# Supplementary material for: A fluorescent hormone biosensor reveals the dynamics of jasmonate signalling in plants
Source: Nat Commun. 2015 Jan 16;6:6043. doi: 10.1038/ncomms7043 (PMC4338584; doi:10.1038/ncomms7043)
Supplement: Supplementary Figures and Table — Supplementary Figures 1-9 and Supplementary Table 1 [file ncomms7043-s1.pdf]

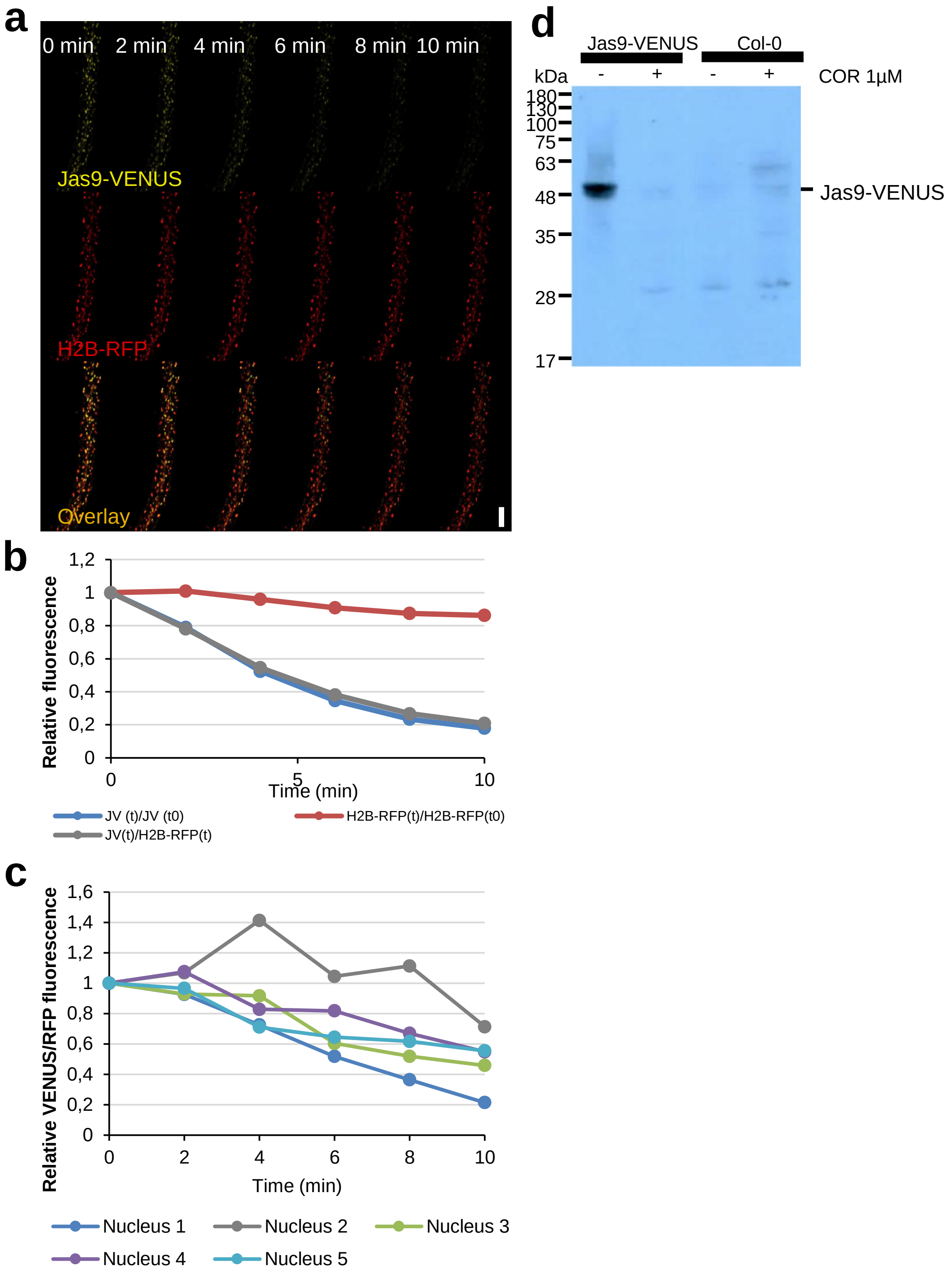

Supplementary Figure 1. Jas9-VENUS but not H2B-RFP is degraded in response to coronatine. (**a**) Time course confocal laser scanning micrographs of Jas9-VENUS, H2B-RFP and the overlay of both signals at the indicated time points after treatment with 1 µM coronatine (scale bar = 100µm). (**b** and **c**) Time course quantification of Jas9-VENUS fluorescence, H2B-RFP fluorescence and of Jas9-VENUS fluorescence normalised to H2B-RFP fluorescence, either measured from all the nuclei visible on the image (**b**) or from randomly selected individual nuclei (**c**). (**d**) Uncropped version of the western blot shown in Figure 1c that shows the analysis of total protein extracts of Jas9-VENUS and Col-0 seedlings treated for 30 minutes with or without 1µM coronatine and probed with an anti-GFP antibody. JV: Jas9-VENUS.

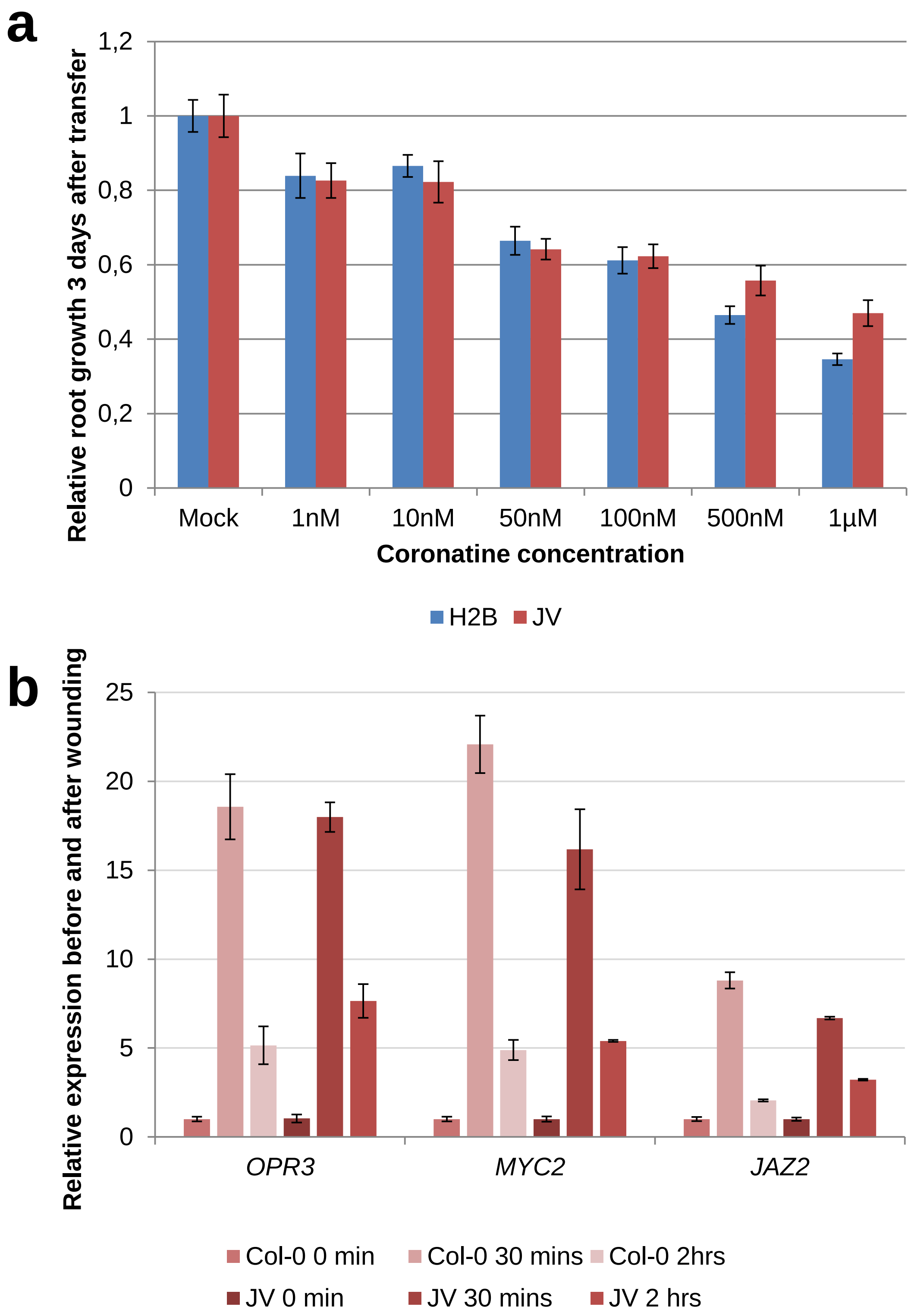

Supplementary Figure 2. The Jas9-VENUS sensor does not affect physiological or transcriptional responses to JA. (**a**) 1-week old H2B-RFP (parental line, control) and Jas9-VENUS seedlings show a similar inhibition of root growth by coronatine. Error bars represent the SEM. (**b**) Shoots and roots of wounded Col-0 and Jas9-VENUS seedlings were harvested 30 minutes and 2 hours after wounding by cutting a cotyledon and the expression of wound inducible genes were investigated using RT qPCR. The genes display a similar dynamic expression pattern in both genetic backgrounds. The experiment was repeated twice and the results show a representative dataset. Error bars represent the standard deviation of four technical replicates. JV: Jas9-VENUS.

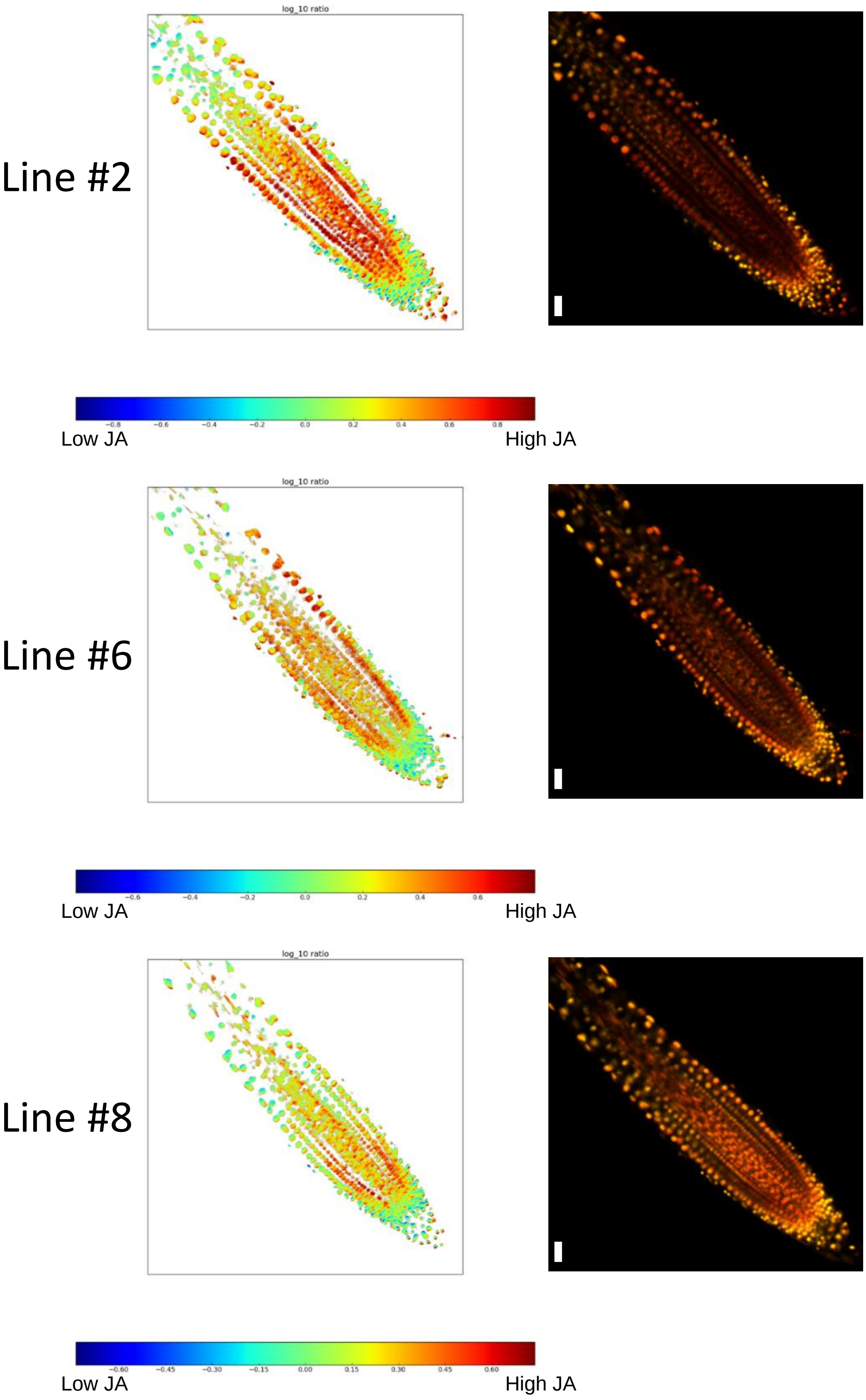

Supplementary Figure 3. Jas9-VENUS root maps of three independent lines. Distribution map of bioactive JA in the root apical meristem based on the ratio of Jas9-VENUS fluorescence to H2B-RFP fluorescence of a representative seedling for three independent transgenic lines imaged using settings chosen to avoid pixels saturation (see Methods for computing details). Similar patterns were observed in the different Jas9-VENUS lines. As the three lines were generated from the same H2B-RFP line, the differences observed between the 3 lines are likely to represent the impact of the transgene insertion site on its expression. The overlay of the Jas9-VENUS and H2B-RFP channels used for generating the root map is shown (scale bar = 30µm).

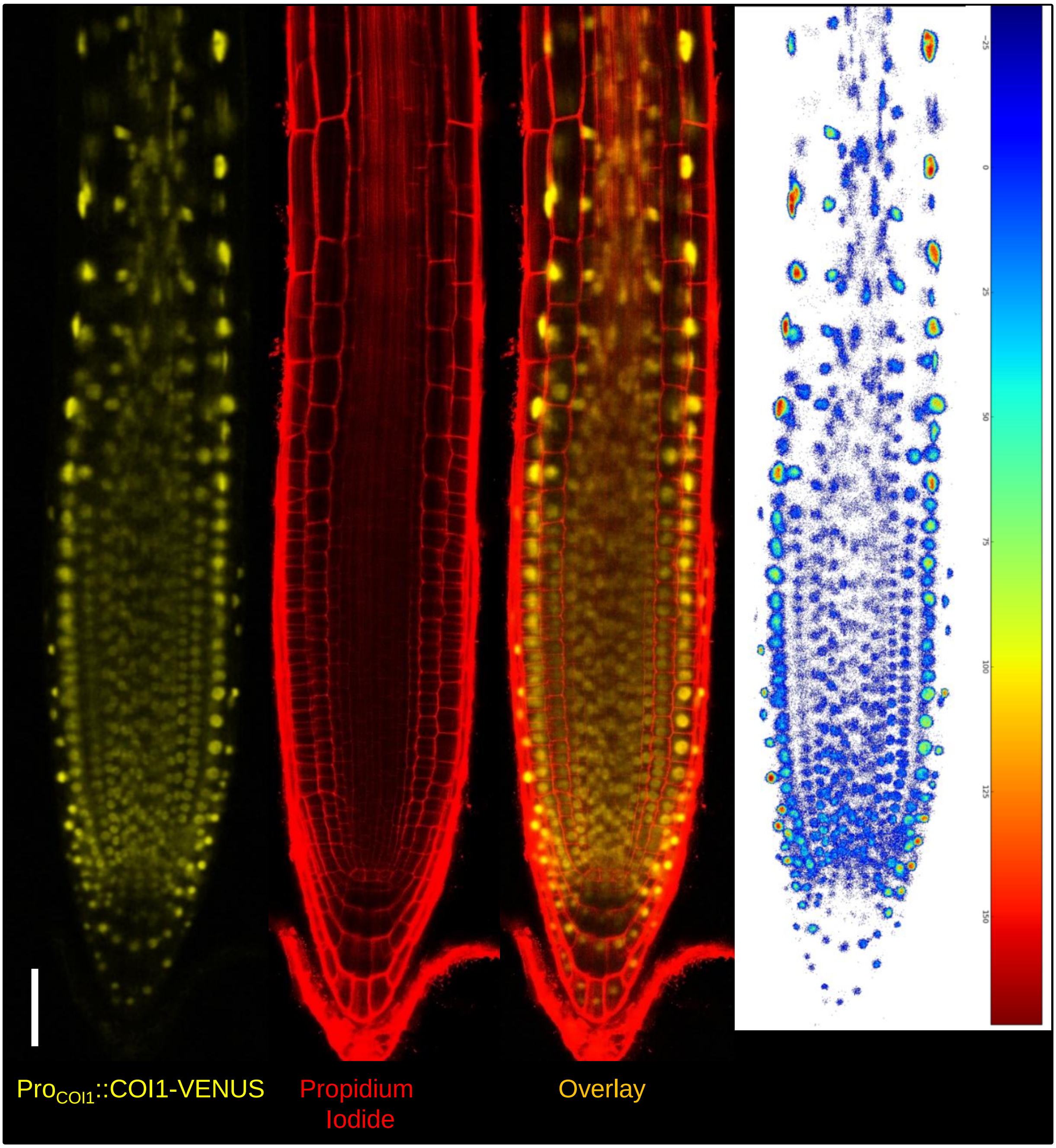

Supplementary Figure 4. COI1-VENUS expression map. Distribution map of proCOI1::COI1-VENUS in the root apical meristem based on the COI1-VENUS fluorescence of a representative seedling of a representative transgenic line. The original image used for generating the root map is shown (scale bar = 30µm) (see Methods for computing details).

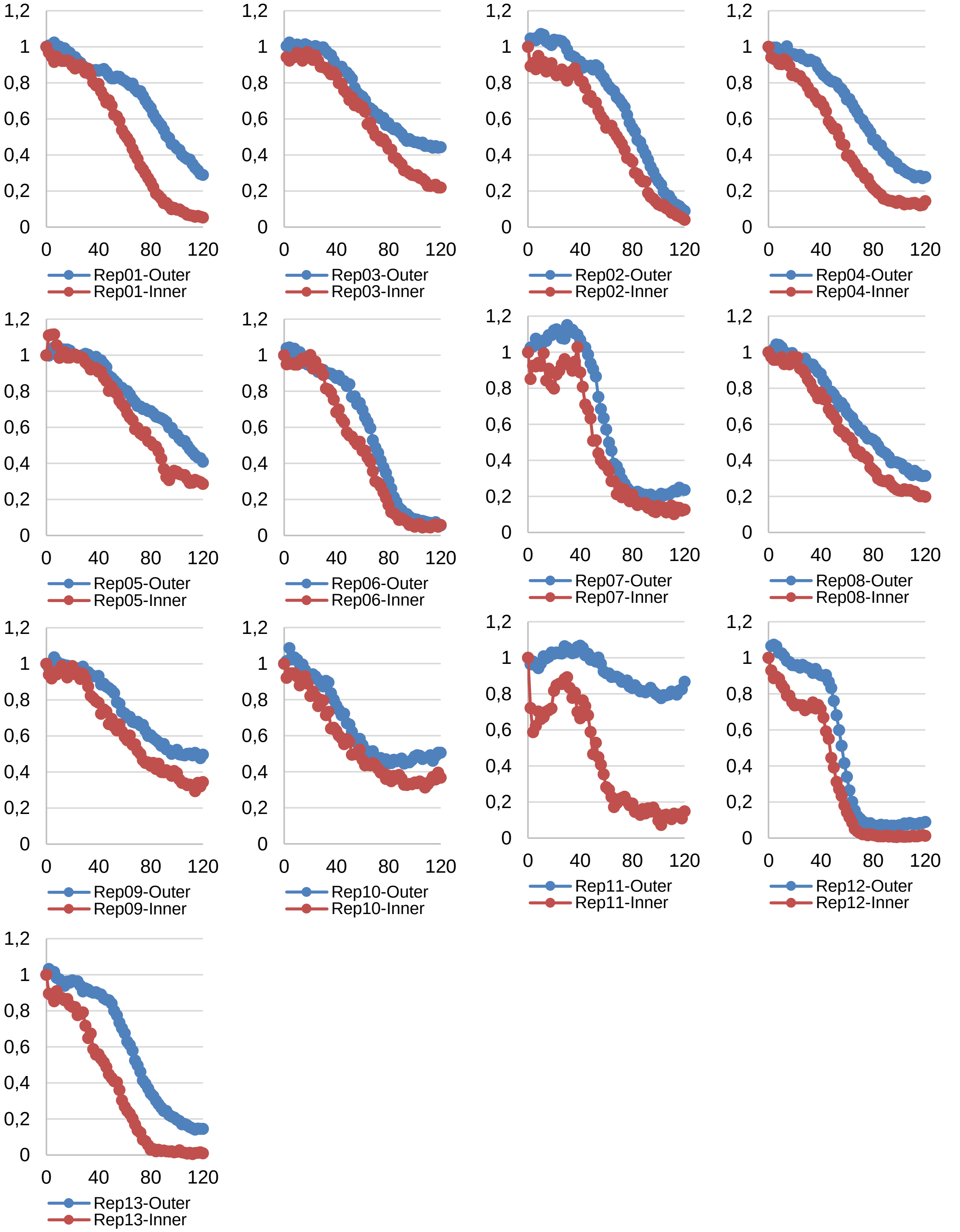

Supplementary Figure 5. Jas9-VENUS degradation in response to wounding is faster in the inner tissues compared to the outer tissues. Seedlings were wounded by cutting a cotyledon using dissection scissors. Time course quantification of Jas9-VENUS fluorescence, normalised to H2B-RFP fluorescence, in the inner and outer tissues for the 13 replicates that were used to generate the averaged quantification shown on Figure 3a. The x-axis shows time in minutes after wounding and the y-axis shows the relative fluorescence.

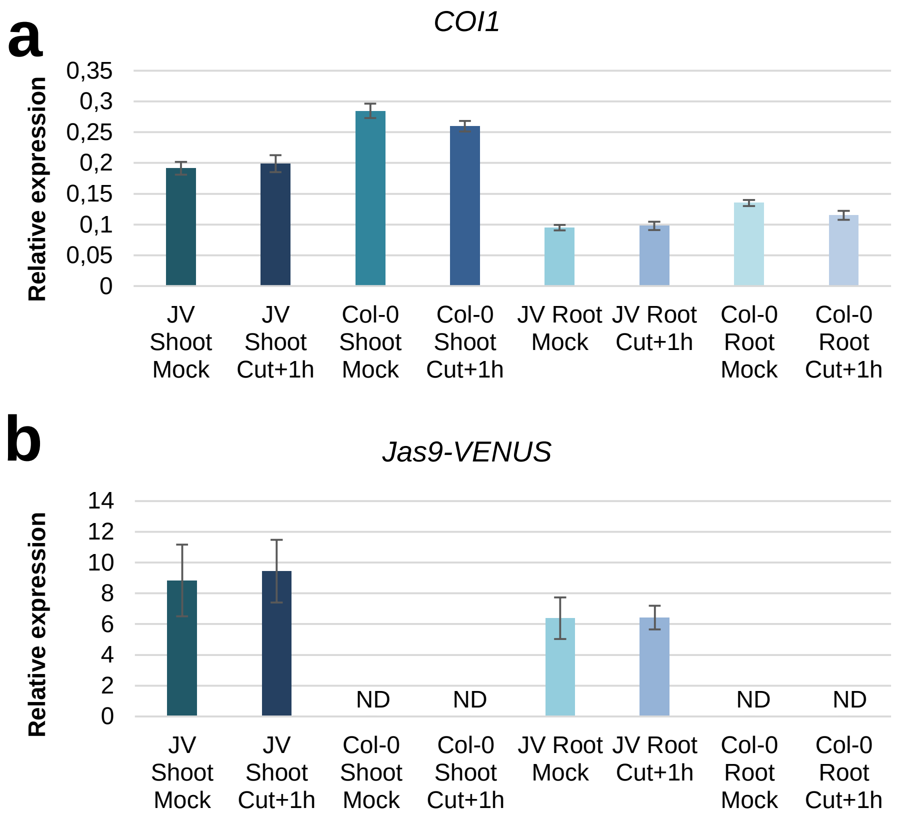

Supplementary Figure 6. Wounding does not alter the expression levels of the JA receptor *COI1* or of the *Jas9-VENUS* sensor. (**a** and **b**) Seedlings were wounded by cutting a cotyledon and shoot and root samples were harvested separately. The expression of *COI1* (**a**) and *Jas9-VENUS* (**b**) were investigated by RT qPCR 1 hour after wounding. The experiment was repeated twice and the results show a representative dataset. Expression of *COI1* and *Jas9-VENUS* were normalised to the expression of the control gene *At1G04850* (CTRL1). Error bars represent the standard deviation of four technical replicates. JV: Jas9-VENUS transgenic line, Col-0: control line, ND: Not Detected.

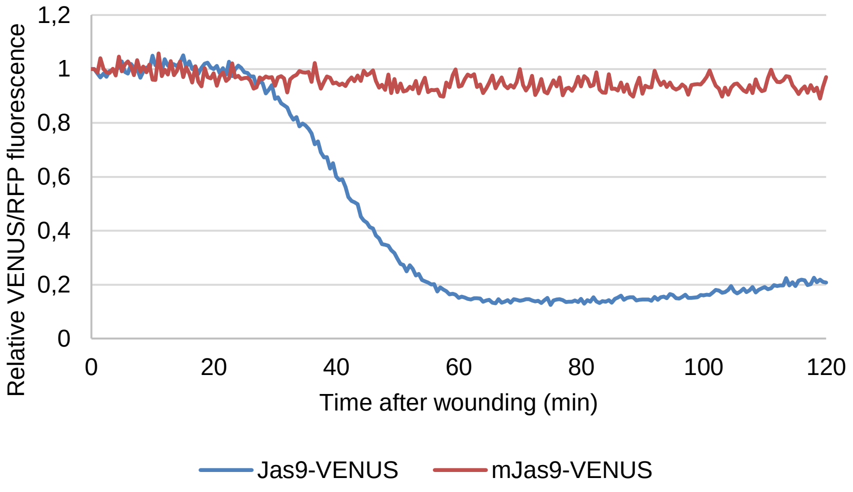

Supplementary Figure 7. Jas9-VENUS degradation in response to wounding requires a functional Jas motif. Seedlings were wounded by cutting a cotyledon using dissection scissors. Jas9-VENUS and Jas9m-VENUS fluorescence, normalised to H2B-RFP fluorescence were quantified on the whole root after wounding for 120 min. The experiment was repeated twice and the results show a representative dataset.

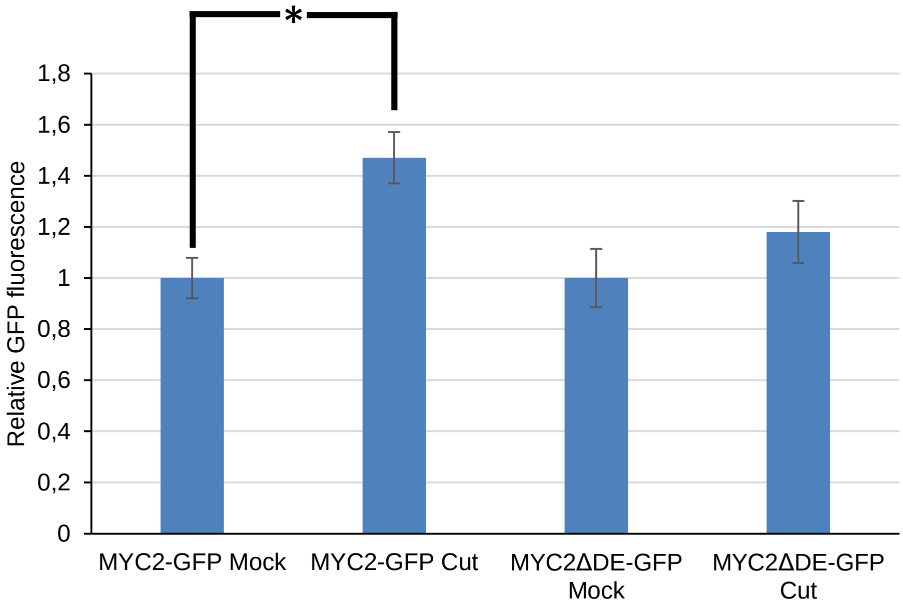

Supplementary Figure 8. MYC2-GFP is stabilized in the root after wounding a cotyledon. 35S:MYC2-GFP and 35S:MYC2ΔDE-GFP seedlings were wounded and the GFP fluorescence was measured two hours after wounding. The GFP fluorescence measured was statistically higher (t-test, α=0.05, P=0.0008) in wounded MYC2-GFP compared to unwounded MYC2-GFP, showing a stabilization of MYC2 protein. The stabilization of MYC2 is not apparent when the Destruction Element (DE) motif is absent since MYC2 is constitutively stabilized (t-test, α=0.05, P=0.27). Error bars represent the SEM.

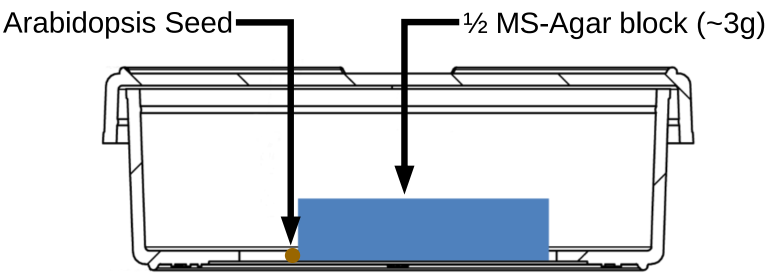

Supplementary Figure 9. Schematic diagram of glass-bottom Petri dishes (base diameter=35mm) used for time course experiments. For treatments and wound response time courses, seeds were sown as shown on the diagram. Original drawing kindly provided by Greiner Bio-One GmbH, Frickenhausen, Germany.

Supplementary Table 1. Primers used in this study.

Cloning primers

qOPR3-F	aagattcgatctctctcatcgagt
qOPR3-R	ggagtggtccgttgagca
qMYC2-F	tccgagtccggttcattct
qMYC2-R	tctcgggagaaagtgttattgaa
qJAZ2-F	ctctttagcctgcgaactcc
qJAZ2-R	ttggtatggtgcctttgatg
qJas9-VENUS-F	tcttgatttgtcgaccggaga
qJas9-VENUS-F	cccggtgaacagctcctc
qCOI1-F	gtgtcctaatttggaagttctcg
qCOI1-R	ctccattccttgttcatctgc
qCTRL1-F	agtggagaggctgcagaaga
qCTRL1-R	ctcgggtagcacgagcttta

qPCR primers

JAZ9-F2	caccatgatcatgttatgcgccgggaacgg
JAZ9-R1	tgtaggagaagtagaagagtaattc
JAZ9-F3m	cctcaagctgctgcggcatccttgg
JAZ9-R3m	ccaaggatgccgcagcagcttgagg
COI1-Fw	caccctcctcgagtgcatcatcaa
COI1-Rev-Full	tattggctccttcaggactc
